# Successful Use of Plasma Exchange in the Treatment of Corticosteroid-Refractory Eosinophilic Granulomatosis with Polyangiitis Associated with Gastrointestinal Manifestations

**DOI:** 10.1155/2016/8341751

**Published:** 2016-02-17

**Authors:** Kohei Tsujimoto, Masato Yagita, Masashi Taniguchi, Masaaki Fujita

**Affiliations:** Department of Clinical Immunology and Rheumatology, Kitano Hospital, The Tazuke Kofukai Medical Research Institute, Osaka 530-8480, Japan

## Abstract

We describe the case of a 33-year-old woman having corticosteroid-refractory eosinophilic granulomatosis with polyangiitis (EGPA) who presented with abdominal pain and responded dramatically to plasma exchange. Eosinophilia, asthma history, neuropathy, pulmonary infiltrates, and paranasal sinus abnormalities confirmed the diagnosis of EGPA. Treatment was initiated with 1 g/day of methylprednisolone pulse therapy for 3 days followed by 60 mg/day of intravenous prednisolone without relieving abdominal pain. Then, plasma exchange was performed thrice. Abdominal pain disappeared after the first plasma exchange. Indication of plasma exchange for EGPA remains controversial; however, it may represent a valid option in cases with gastrointestinal involvement.

## 1. Introduction 

Eosinophilic granulomatosis with polyangiitis (EGPA), previously termed Churg-Strauss syndrome (CSS), is defined as vasculitis affecting small- and medium-sized arteries. EGPA is characterized by allergic rhinitis, asthma, and eosinophilia and may also involve several visceral organs including the lung, heart, kidney, and gastrointestinal (GI) tract [1]. GI involvement is observed in approximately 20%–30% of EGPA patients and may be life-threatening in cases of perforation [1]. Severe and specific GI manifestations are considered as poor prognostic indicators and represent one of the components of the Five-Factor Score (FFS), commonly used to determine the prognosis of EGPA [2].

Adjunct plasma exchange is widely used as a rescue therapy in life-threatening situations. However, there is a lack of consensus regarding the optimal plasma exchange protocol for the treatment of EGPA. Further, there is significant geographical variability in the indications, duration, frequency, and number of sessions of plasma exchange. The majority of centers use plasma exchange in cases of severe nephritis and lung hemorrhage [3–5]. However, the efficacy of plasma exchange for GI involvement has yet to be demonstrated. Herein, we present a case of a patient with corticosteroid-refractory EGPA with GI manifestations who responded dramatically to plasma exchange.

## 2. Case Report

**Figure 1:**
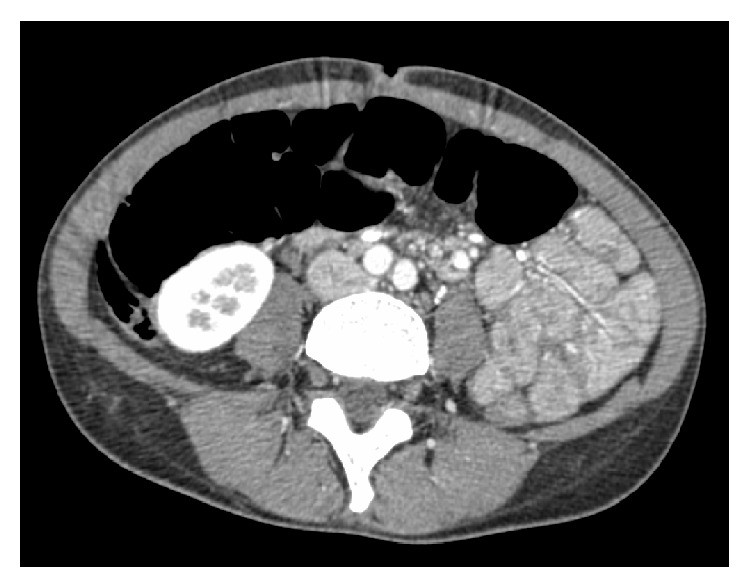
Contrast-enhanced abdominal computed tomography demonstrating small intestine wall thickening with fluid retention.

**Figure 2:**
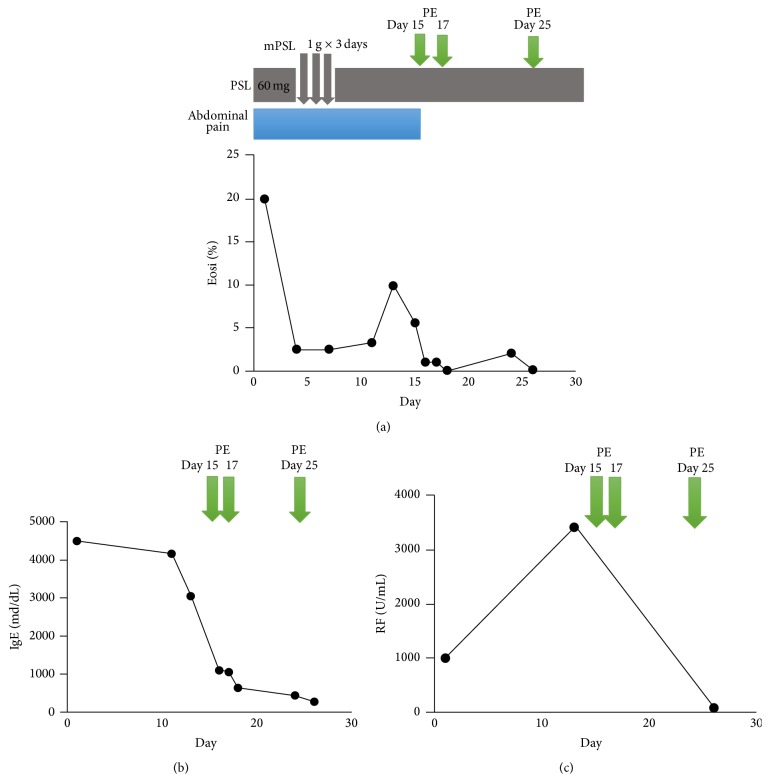
Clinical course of the present case. (a) Treatment timing and trends in eosinophil count. (b), (c) The effect of plasma exchange on IgE and RF. PSL, prednisolone; Eosi, eosinophil; RF, rheumatoid factor.

A 33-year-old woman with a history of asthma was hospitalized because of a 4-week history of intermittent abdominal pain in the epigastric region, nausea, and numbness of the distal extremities. There were no episodes of diarrhea or bloody feces. The patient had no previous gastrointestinal history. On admission, the patient was afebrile with normal blood pressure. Physical examination showed tenderness in the epigastric region and diminished bowel sounds. There were no rashes, arthritis, or oral ulcers. Lung and cardiac auscultation revealed no abnormalities. Any neurological disorder was not observed objectively, although the patient complained of numbness of the distal extremities. The laboratory test results are shown in Table 1 (e.g., IgE, 4500 mg/dL; Eos, 3810/*μ*L (20%)). Both PR3-ANCA and MPO-ANCA were negative. Anti-nuclear, anti-ds-DNA, anti-cardiolipin, and anti-glomerular basement membrane autoantibodies were all negative. Anti-hepatitis B virus core antibody** (**HBc-Ab), anti-hepatitis B surface antibody** (**HBs-Ab), and hepatitis B surface antigen (HBs-Ag) were all negative. Anti-hepatitis C virus antibody was negative. Cryoglobulin studies were not performed. CHIC2 (4q12) deletion, which is consistent with FIP1L1-PDGFRA fusion, was not observed using fluorescent in situ hybridization. Chest computed tomography (CT) revealed bilateral ground glass opacities at the lung bases. Contrast-enhanced abdominal CT demonstrated small intestine wall thickening with fluid retention (Figure 1). A gastroscopy showed superficial gastritis and gastroesophageal reflux disease-** (**GERD-) Grade M. A colonoscopy showed no abnormalities. Esophageal, gastric, and duodenal biopsies revealed no evidence of inflammatory eosinophil infiltration. Neurophysiological examination revealed a low F-wave frequency affecting the left ulnar nerve and slightly decreased peroneal compound muscle action amplitude in the left peroneal nerve. Bone marrow examination revealed increased numbers of megakaryocyte but no evidence of dysplasia. Contrast-enhanced magnetic resonance imaging revealed the presence of polyps in the left maxillary sinus with no brain abnormalities. Electrocardiogram and cardiac ultrasonography results were normal. The patient fulfilled the 1990 American College of Rheumatology classification for Churg-Strauss syndrome [6] according to the following criteria: asthma, >10% increase in eosinophil numbers, neuropathy, pulmonary infiltrates, and paranasal sinus abnormalities. She was diagnosed with EGPA/CSS 6 weeks after the onset of gastrointestinal manifestations. Treatment was initiated with 1 g/day methylprednisolone pulse therapy for 3 days followed by 60 mg/day intravenous prednisolone (equivalent to 40 mg/day PO), which led to improvements in the numbness of the distal extremities and almost complete resolution of the pulmonary infiltrative shadow. CRP became normal. However, abdominal pain persisted during corticosteroid therapy and serum IgE levels, serum RF levels, and eosinophil counts remained high (IgE, 3050 mg/dL; RF, 3430 IU/mL; and Eos, 549/*μ*L (5.6%)). GI manifestation of the case was considered as corticosteroid resistance. Therefore, additional immunosuppressive therapy was required for the management of GI manifestation. However, the patient denied administration of cyclophosphamide because she had the desire to bear children. As one month has passed since she complained of abdominal pain, plasma exchange was performed in an expectation of immediate effect. We followed the general protocol used for malignant rheumatoid arthritis in Japan. At first, we planned to perform plasma exchange four times; however, the symptoms or laboratory results became normal and we stopped it at the third time. Albumin was used as the replacement solution at the first time and fresh frozen plasma at the second and third time. Estimated plasma volume (in liters) was calculated as follows: body weight (kg) × (1 − hematocrit)/12. After the first plasma exchange, abdominal pain completely resolved and IgE decreased to 1090 mg/dL and eosinophils decreased to 112/*μ*L (1.0%). As the neuropathy was improved before plasma exchange, the effect of plasma exchange on the neuropathy could not be evaluated. After the third plasma exchange, IgE level became normal and RF decreased to 80 IU/mL. She was discharged from the hospital in six weeks (Figure 2). Prednisolone was decreased to 10 mg/day in 6 months without any recurrence, including abdominal symptoms. The follow-up imaging studies were not performed.

## 3. Discussion

EGPA is typically corticosteroid responsive with prompt treatment [7]. However, some patients develop severe complications, such as bowel perforation, infarction, or obstruction [7]. Additional immunosuppression is recommended for patients with severe or corticosteroid-resistant EGPA to avoid these life-threatening complications. Although intravenous cyclophosphamide (CY) is often administered as first additional immunosuppressive drug, CY is associated with long-term side effects, including infertility and premature menopause in young women [8], and the administration of CY is commonly avoided in young women. In our case, the patient denied the administration of CY. As one month had passed since she complained of abdominal pain and the situation was life-threatening, we decided to perform plasma exchange instead of other immunosuppressive agents, such as rituximab and mycophenolate mofetil, in an expectation of immediate effect.

Adjunct plasma exchange is widely used as rescue therapy in life-threatening situations. However, the utility and indication of plasma exchange in EGPA have remained controversial for the last three decades. Guillevin et al. reported two randomized controlled trials in patients with a diagnosis of EGPA or periarteritis nodosa in the 1990s [9, 10]. Neither study reported statistical improvements in disease control or survival with the addition of plasma exchange. However, these two studies included heterogeneous populations. In addition to cases of EGPA, patients with periarteritis nodosa were also enrolled. Moreover, patients with renal or lung involvement, in addition to those with GI manifestations, were included in these studies. A careful evaluation of the indications of plasma exchange in subgroups of patients with GI involvement has to be performed [11].

Widespread activation of the Th-2 cellular-mediated inflammatory response and humoral immunity are believed to play important roles in the pathogenesis of EGPA. All types of vasculitis have the potential to cause pathological changes in the GI tract as a result of vessel walls inflammation leading to alterations in blood flow and ischemic change [7]. The general principal of plasma exchange relies on the removal of various substances from the blood, including antibodies and immune complexes. Further, plasma exchange improves the capacity of the reticuloendothelial system to clear immune complexes [9]. Based on this mechanism, plasma exchange represents a valid option for the treatment of EGPA patients, particularly those with elevated serum RF or IgE levels indicating the involvement of the humoral immune system. In the present case, the elevated serum RF and IgE levels were promptly decreased, and GI manifestations were improved in response to plasma exchange. Although the possibility that plasma exchange improved the small intestinal edema through removal of some fluid as dialysis could not be ruled out, these findings support our original hypothesis that plasma exchange has efficacy in the subgroups of patients with specific EGPA symptoms such as GI manifestations.

The limitation of this study is a lack of direct evidence supporting the efficacy of plasma exchange. First, we could not identify the causative agents affecting GI manifestations of EGPA, which was removed by plasma exchange. Second, corticosteroids alone may have led to remission in this case, as corticosteroids were not stopped. However, the dramatic change after first plasma exchange should not be ignored. The fact that an 8-week history of abdominal pain disappeared immediately after the first plasma exchange suggests plasma exchange improved GI manifestations.

In many cases, prednisolone and immunosuppressive agents have substantial efficacy in the treatment of EGPA making the routine use of plasma exchange unnecessary. However, to avoid life-threatening complications, plasma exchange may be considered in cases of severe or corticosteroid-resistant EGPA in an expectation of immediate effect, even in patients without renal or lung hemorrhage. The findings of the present case indicate that plasma exchange represents a valid therapeutic option in EGPA patients with GI involvement. Intravenous immunoglobulin (IVIG) was not administrated in this case. However, Tsurikisawa et al. demonstrated the efficacy of IVIG for patients with both cardiac and neurological manifestations [12]. Also Danieli et al. reported long-term effectiveness of IVIG synchronized with plasma exchange in patients with EGPA, although the beneficial effects of the addition of plasma exchange were unclear [13]. IVIG may also be another valid option for corticosteroid-resistant EGPA.

## Figures and Tables

**Table 1:** 

Hematological test			
Hb	11.1 g/dL	IgE	4500 mg/dL
WBC	19,500/*μ*L	IgG	1520 mg/dL
Neu	14300/*μ*L (73.4%)	IgG4	295 mg/dL
Lymph	897/*μ*L (4.6%)	RF	996 IU/mL
Mono	702/*μ*L (3.6%)	Troponin-I	<0.015 ng/mL
Eos	3810/*μ*L (20%)	MPO-ANCA	<1 U/mL
Baso	38/*μ*L (0.2%)	PR3-ANCA	1.5 U/mL
Plt	454 × 10^3^/*μ*L		
CRP	3.38 mg/dL		
BUN	8.9 mg/dL		
Cre	0.48 mg/dL		
